# Insight into *Kytococcus schroeteri* Infection Management: A Case Report and Review

**DOI:** 10.3390/idr13010026

**Published:** 2021-03-14

**Authors:** Shelly Bagelman, Gunda Zvigule-Neidere

**Affiliations:** 1 International Students Department, Riga Stradins University, LV-1007 Riga, Latvia; 2 Department of Pediatrics, Riga Stradins University, LV-1007 Riga, Latvia; Gunda.zvigule-neidere@rsu.lv

**Keywords:** *Kytococcus*, *Schroeteri*, antibiotic resistance, antibacterial therapy, resistance

## Abstract

Background: *Kytococcus schroeteri* is a member of normal skin microflora, which can cause lethal infections in immunosuppressed hosts. In this review we attempted to draw patterns of its pathogenicity, which seem to vary regarding host immune status and the presence of implantable devices. Evidence suggests this pathogen houses many resistance-forming proteins, which serve to exacerbate the challenge in curing it. Available information on *K. schroeteri* antibacterial susceptibility is scarce. In this situation, a novel, genome-based antibiotic resistance analysis model, previously suggested by Su et al., could aid clinicians dealing with unknown infections. In this study we merged data from observed antibiotic resistance patterns with resistance data demonstrated by DNA sequences. Methods: We reviewed all available articles and reports on *K. schroeteri*, from peer-reviewed online databases (ClinicalKey, PMC, Scopus and WebOfScience). Information on patients was then subdivided into patient profiles and tabulated independently. We later performed *K. schroeteri* genome sequence analysis for resistance proteins to understand the trends *K. schroeteri* exhibits. Results: *K. schroeteri* is resistant to beta-lactams, macrolides and clindamycin. It is susceptible to aminoglycosides, tetracyclines and rifampicin. We combined data from the literature review and sequence analysis and found evidence for the existence of PBP, PBP-2A and efflux pumps as likely determinants of *K. schroeteri*. Conclusions: Reviewing the data permits the speculation that baseline immune status plays a role in the outcome of a *Kytococcal* infection. Nonetheless, our case report demonstrates that the outcome of a lower baseline immunity could still be favorable, possibly using rifampicin in first-line treatment of infection caused by *K. schroeteri*.

## 1. Introduction

The *Kytococcus* genus was first distinguished from the Micrococcus species in 1995 [1]. It is now considered to belong to the family *Dermacoccaceae* and order *Actinomycetales*. Researchers described it as Gram-positive, non-encapsulated, and non-motile bacteria. *Kytococci* are aerobic, catalase-positive bacteria that form yellow colonies on agar [2]. The genus is now known to include three species, *K. schreoteri, K. sedentarius* and *K. aerolatus*, though the taxonomy browser of NCBI reports more unclassified and uncultured variants [3]. *K. schroeteri* is a natural and common inhabitant of human skin flora.

Infections with *Kytococcus schroeteri* are uncommon (only 20 publications in the last 17 years [4,5,6,7,8,9,10,11,12,13,14,15,16,17,18,19,20,21,22]), and since the species itself has only recently been discovered, the information that is now available for physicians remains scarce. Treatment encompasses two main challenges—a difficult identification process and consistent antibiotic resistance. Based on this literature review, it appears that *K. schroeteri* requires an immunodeficient host or an implanted device to adhere to, or a combination of the two.

When antibiotic sensitivity data is lacking, and reports of clinical success stories are limited, whole-genome sequencing for antibiotic susceptibility testing (WGS-AST) is now a powerful alternative [23]. By combining the experience of previously published cases, sequence analysis and the knowledge we have recently gained from successfully treating an infant with *Kytococcus* sepsis, we now hope to present a clearer approach to treating this rare and sometimes deadly infection.

This novel model aims to create effective treatment plans by shifting the order—using bacterial genome sequence data to predict resistance to antimicrobials, rather than using classic antibiotic susceptibility tests.

## 2. Methods

We performed a literature review based on all available studies and reports on *K. schreoteri* infection (22 in total), including our own recent experience with this pathogen in the Children’s Clinical University Hospital in Riga, Latvia. Of the identified bibliographic references, only relevant online publications in peer-reviewed journals accessible through ClinicalKey, PubMed, Scopus and Web of Science were retrieved for further analysis. Due to scarce reports, the inclusion criteria for this review were broad and included all existing reports of *K. schreoteri* infections. All publication dates and geographical locations were included. Both adults and pediatric populations as well as all patient outcomes were included. Exclusion criteria were non-human subjects. Information on patients was subdivided into patient profiles (immunocompromised, orthopedic prosthetics-implanted, pediatric associated with implanted devices) and tabulated independently. References of included publications were manually screened for additional studies. For all case report publications referenced in this review, antibiotic resistance analysis was performed and tabulated (Table 1).

*K. schreoteri* whole genome shotgun sequences from strain H01 (GenBank: VHHR00000000.1) [24,25] and strain UMB1298 (NCBI Reference Sequence: NZ_PKIZ00000000.1) [26] were analyzed with the Comprehensive Antibiotic Resistance Database (CARD) algorithm. The Resistance Gene Identifier was used to predict resistome patterns of *K. schreoteri*.

Parental consent was obtained for the case report, and the Riga Stradins University Ethics Committee reviewed and confirmed the study.

## 3. Case Report

A nine-month-old infant, recently diagnosed with Acute Myeloid Leukemia (AML) and treated according to the NOPHO-DBH AML-2012 protocol, presented with neutropenic fever. Laboratory tests showed bone marrow aplasia, neutropenia (0.01 × 10^3^/μL) and thrombocytopenia (17 × 10^3^/μL), and she was started on empiric treatment of ceftazidime (50 mg/kg/dose) and amikacin (15 mg/kg).

The patient presented signs of sepsis and was admitted to the ICU with a neutrophil count of 0.00 × 10^3^/μL and severe nosocomial rotavirus gastroenteritis, both of which seemed to contribute significantly to the patient’s deteriorating condition. After obtaining results of blood and Port-a-cath cultures, which demonstrated a *K. schreoteri* infection (using MALDI-TOF MS), ceftazidime was switched to meropenem (10 mg/kg), based on a targeted literature search at the time. Meropenem was substituted for vancomycin (60 mg/kg/day), based on in vitro sensitivity data, though it was difficult to establish whether the bacteria truly are vancomycin-sensitive due to lab limitations. Clinically it seemed that the patient did not respond to vancomycin as expected, supporting the suspicion that the bacteria is not vancomycin susceptible.

The patient was transferred from the ICU to the hematooncology ward, where the Port-a-cath was evacuated due to local infiltration. The patient remained febrile, and meropenem was re-administered. Due to persistent neutropenia and a maculopapular rash, amikacin was substituted by rifampicin (20 mg/kg). The rash was suspected to be of fungal origin, though there is data supporting *Kytococcus*-related maculopapular rashes [7]. Following clinics of cough and desaturation, a lung CT was performed and demonstrated bilateral infiltrate. Voriconazole (3–4 mg/kg) was added to the treatment regimen. Finally, on day 23 the patient was afebrile, and a significant improvement in cell counts was noted.

## 4. Results

Based on this literature review, *K. schreoteri* infections appear to manifest in two major forms: bacteremia (Table 2) and implant device-associated (Table 3).

Table 2 shows that seven of the eight cases of bacteremia and pneumonia were associated with an underlying malignancy.

It appears that immune status played a more prominent role than both definitive and empirical treatments in bacteremia cases.

The cases that involved an implanted device are subdivided in Table 3, Table 4 and Table 5.

As shown in Table 3, an artificial valve was implanted (mostly years before infection) in all endocarditis cases. Three of the eight cases underwent surgery for replacement (and if needed, debridement) of the affected valves. All cases recovered. Table 4 demonstrates that among orthopedic-prosthetic adult cases, there were reports of an underlying condition in two of the three cases. Cases underwent surgery and recovered after antimicrobial therapy. All three pediatric cases in Table 5 had an implanted ventriculoperitoneal shunt. Two of the three cases underwent surgery; these were also the cases that recovered. One case was deceased. Comparison of the survival ratios between the two groups suggests that immunosuppression could play a major role in recovery from this infection. Six of eight bacteremia patients died (Table 2), in comparison to one of 14 implant-device patients who died (Table 3, Table 4 and Table 5). However, only two of the eight bacteremia cases received rifampicin, compared to six out of the eight endocarditis patients who received it. Resistance to antibiotics described by case reports is shown in Table 1, though not all case reports described results for all antibiotic groups.

*K. schreoteri* whole genome shotgun sequences from strain H01 (GenBank: VHHR00000000.1) and strain UMB1298 (NCBI Reference Sequence: NZ_PKIZ00000000.1) were analyzed with the CARD algorithm. Both strains had 91 matching Antibiotic Resistance (AMR) genes; however, these were only by loose hits. According to the definition by CARD, these correspond to the criteria of more distant homologs of AMR genes. Both strains showed similarities with several vancomycin resistance gene clusters: *vanG* (38.25%) and *vanTG* (27.86%), *vanI* (23.13%) and *vanWI* (25.21%), and *vanHB* (32.46%) and *vanXYC* (29.94%). *K. schroeteri* has Penicillin Binding Protein-2 (PBP-2, PBP-2a) (mecA,B,C: 27.39%, 28.45% and 22.81%, respectively), which explains penicillin resistance.

## 5. Discussion

In 2002, *K. schreoteri* was described by Becker et al., after culturing the blood of a 34-year-old woman diagnosed with endocarditis [2]. The final identification was attained by determining the 16S rDNA sequence, which was 97.9% similar to that of *K. sedentarius* (identified previously in 1944, ZoBell and Upham [24]). However, the DNA-DNA hybridization allowed for classification of the newly described bacterium into an independent genospecies, as it did not reach the threshold of delineation to *K. sedentarius* (45.4%) [2].

There appear to be several issues regarding the correct identification of this pathogen [27]. As they are common skin microflora, the identification of *K. schreoteri* in culture could be mistaken for mere contamination, and not all automated devices that are currently used in laboratories worldwide consider the *Kytococcus* species. The common pathway that led to the correct identification of *K. schroeteri*, undertaken by almost all physicians in the studies presented in this review, consisted of three steps: automated system (VITEK2, BD Phoenix or API), biochemical testing, and sequencing. As *Kytococcus* is a rarely encountered pathogen, identification is often achieved by using MALDI-TOF MS. At the moment, there is no specialized growth media required, as the bacterium easily grows on blood agar. There is also no standardized disc diffusion data to attain antibiotic susceptibility testing. The strands used for WGS-AST in this review were taken from GenBank and not from our hospital’s laboratory; thus, there are no practical methods that could support the resistance we matched with CARD.

*K. schreoteri* is resistant to penicillin, methicillin, oxacillin, cephalosporins, erythromycin and clindamycin. Resistance to fluoroquinolones (ciprofloxacin, ofloxacin) varies when comparing the data from literature analysis and CARD (Table 1).

The resistance to penicillin is well established and consistent across all studies reviewed. The *Kytococcal* genome houses a number of genes that code for resistance proteins, mainly PBP-2 and PBP-2a, which could explain, with high probability, its resistance to penicillin and methicillin [28].

*mef(B)* [29]), *oleC* [30] and *oleB* [31] matched with 26.3%, 38.5% and 35% similarity to the two strains, respectively. These genes code for ATP-Binding Cassette (ABC) transporters, which function as efflux pumps for macrolide group antibiotics. This supports the data we obtained from the literature review, demonstrating *K. schroeteri’s* possible resistance mechanism to macrolides.

*novA*, a type III ABC transporter [32], identified on the novobiocin biosynthetic gene cluster, matched by 33.4% with the strains discussed.

*AbaQ* belongs to the Major Facilitator Family (MFC) [33], and *mdtK* is a part of the subfamily of the multidrug and toxic compound extrusion (MATE)-like proteins [34]. *AbaQ* and *mdtK* are transporters, matching with 40.3% and 25.02% similarity to our strains, respectively. The literature review suggests some resistance exists; thus, we would recommend obtaining resistance cutoff points before administration.

Data regarding vancomycin are conflicting. Sensitivity analysis from the literature review (Table 1) suggests it is mostly susceptible; however, both *K. schroeteri* strains demonstrate vancomycin resistance. CARD analysis showed distant homology (loose hits) to several vancomycin resistance genes. *Van I* gene, which codes for D-Ala--D-Ala ligase, is responsible for the biosynthesis of alternate cell-wall precursors in bacteria that are resistant to vancomycin [35]. *vanWI*; *VanG*, which are D-Ala-D-Ala ligase homologs that can synthesize D-Ala-D-Ser, are an alternative substrate for peptidoglycan synthesis, which reduces vancomycin binding affinity [36]. *vanTG* (a *vanT* variant) is found in the *vanG* gene cluster. Combined with our clinical experience, which showed a reduced response to vancomycin, these data raise the question of vancomycin’s place in the treatment regimen and further substantiate the need for sequencing and standardization of *Kytococcal* resistance data.

Though most publications report susceptibility to tetracycline, CARD analysis shows resistance genes. *tetAB(48)* is an efflux ABC transporter [37] of tetracycline antibiotics. As seen in Table 1, the strains of *K. schroeteri* described in this review house *tetA(58)*, which matched with 37.5% similarity to the (48) variant. *otrA* [38] is part of a gene family that functions through ribosomal protection, as tetracyclines inhibit protein synthesis. There was no data on resistance from clinical cases discussed in this review.

*K. schreoteri*’s *rpoB* gene does not harbor any variant associated with high probability of rifampicin resistance, which coincides with our clinical observation. It is also susceptible to gentamycin, chloramphenicol and daptomycin. Clinical breakpoints are not established yet, and several publications relied on the coagulase-negative *Staphylococcus* criteria (after obtaining Gram-positive data) to adjust their treatment plan.

The overview of *Kytococcal* resistance patterns allows us to suggest that this pathogen is sensitive to antibacterial agents that target RNA synthesis, DNA synthesis, folic acid synthesis and protein synthesis (30S subunit).

*K. schreoteri* causes a rare opportunistic infection in two major groups of patients: immunocompromised, for whom bacteremia is mostly lethal, and patients after implant surgery (orthopedic or heart valve), who have a significantly lower mortality. In our case report, as well as in almost all cases depicted in Table 2, blood cultures were preformed alongside bronchoalveolar lavages, sputum cultures, skin and soft tissue biopsies and cultures, and shunt and Port-A-Cath tip cultures. In most cases significant colonization was attributed to *K. schreoteri*, which excludes more common pathogens. We aim to demonstrate that it is *K. schroeteri* that causes these infections in immunocompromised patients. Most likely, the causative organisms in the cases of endocarditis and neutropenic fever differ; therefore, empiric regimens vary. Both the American Heart Association [39] and the European Society Of Cardiology [40] support triple drug therapy for mechanical or native valve endocarditis, and this combination includes rifampicin. The regimen for treating neutropenic fever, however, is based on the third generation cephalosporins for coverage of Gram-negative pathogens. Both bacteremia cases and endocarditis cases received vancomycin empirically; however, broad-spectrum treatment in bacteremia cases was started as well. *K. schreoteri* seems to fall between the cracks, usually until sequencing is performed. As for the orthopedic group, empirical data was not specified, nor were any of the cases treated with rifampicin; perhaps it can be assumed that, being limited to skeletal tissue, this infection is less likely to cause systemic manifestations that might deteriorate to death.

Currently, there are no standardized sensitivity data for *K. schreoteri*; in our case, it seems that the administration of rifampicin was the major contributing factor to the recovery of our patient. This assumption is further substantiated by review of the endocarditis cases, demonstrating a higher favorable outcome rate; the endocarditis protocol includes rifampicin as primary, standard therapy. Alongside antibiotic treatment, we highly recommend the immediate removal of all indwelling devices, as in many of the case reports described in this review, they were colonized upon cultivation and are possibly the biggest threat to the spread of this infection, especially in the immunocompromised population. This could have been the reason for the successful outcome of the *K. schreoteri* infection in those cases.

Limitations of this review arise due to lack of available resistance information, due to which we used two published sequences of the bacteria. As the bacterial intraspecies genome varies, the conclusions we present here might not apply broadly.

Further research is needed to better establish resistance patterns and set MICs that apply to all antibiotics. Multidrug efflux pumps seem to play a role in this bacterium [41] and could be important leads to establish any definitive data. As long as standardized sensitivity remains in question, we suggest that future healthcare providers who find themselves dealing with this peculiar pathogen take into account patient risk factors such as poor immune status and implant devices (which may serve as vehicles or primary sources for biofilm formation). We believe the early administration of rifampicin might result in a favorable outcome.

## Figures and Tables

**Table 1:** Analysis of antibacterial activity.

	Literature Review			CARD Data
Antibiotic Group	Resistant	Susceptible	No Data	Resistance Genes
Beta lactams				
Penicillins	15			*PBP-2*, *PBP-2A*
Cephalosporins	10			*AIM-1*
Carbapenems		7		
Macrolides	12		10	*mef(B)*, *oleC*, *oleB*
Aminoglycosides	1	13	8	
Sulfonamides	1	1	20	
Fluoroquinolones	3	6	13	*AbaQ*, *mdtK*
Rifampicin		14	8	
Vancomycin		15	7	*vanI*, *vanWI*, *vanG*, *vanTG*
Clindamycin	7	1	14	
Daptomycin		3	19	
Linezolid		9	13	
Tetracycline				*otrA*, *tetA(58)*
Novobiocin				*novA*

**Table 2:** Immunosuppressed cases.

Case	Age/Sex	Condition	Underlying Disease	Implanted Device	Therapy	Outcome
Mohammedi et al., 2004 [4]	71 M	Pneumonia, bacteremia	Asthma	-	Ceftriaxone Ofloxacin	Deceased
Hodiamont et al., 2010 [5]	40 M	Pneumonia, bacteremia	AML	-	Vancomycin Rifampin Gentamicin	Deceased
Hodiamont et al., 2010 [5]	52 M	Pneumonia, bacteremia	AML	CVC (Central venous catheter)	Vancomycin Ceftazidime	Deceased
Blennow et al., 2011 [6]	43 F	Pneumonia, bacteremia	AML	-	Vancomycin TZP (Piperacillin/tazobactam) Meropenem Linezolid Trimethoprim/sulfamethoxazole	Recovered
Nagler et al., 2011 [7]	68 M	Skin rash, pneumonia, bacteremia	AML	-	Vancomycin	Deceased
Amaraneni et al., 2015 [8]	50 M	Pneumonia, bacteremia	Hairy Cell Leukemia	CVC	Vancomycin TZP Levofloxacin	Deceased
DeMartini et al., 2016 [9]	17 M	Bacteremia AKI	Myelodysplastic syndrome	-	Glycopeptide Carbapenem	Deceased
Our case	9 m F	Skin rash, pneumonia, bacteremia	AML	CVC	Meropenem Rifampicin Voriconazole	Recovered

**Table 3:** Endocarditis cases.

Case	Age/Sex	Condition	Underlying Disease	Implanted Device	Definitive Therapy	Outcome
Becker et al., 2003 [2]	34 F	Endocarditis	-	Mechanical aortic valve	Vancomycin Rifampin Gentamycin	Recovered
Le Brun et al., 2005 [10]	73 M	Endocarditis	-	Bioprosthetic aortic valve	Surgery Teicoplanin Rifampin Gentamycin	Recovered
Mnif et al., 2006 [11]	49 F	Endocarditis	-	Artificial mitral valve	Surgery Rifampin Pristinamycin	Recovered
Aepinus et al., 2007 [12]	38 F	Endocarditis	Diabetes Mellitus type 1	Mechanical aortic valve	Vancomycin Rifampin Gentamycin Levofloxacin	Recovered
Renvoise et al., 2007 [13]	70 M	Endocarditis	-	Bioprosthetic aortic valve	Surgery Vancomycin Gentamycin	Recovered
Poyet et al., 2010 [14]	72 F	Endocarditis	-	Mechanical aortic valve	Vancomycin Rifampicin Gentamycin	Recovered
Yousri et al., 2010 [15]	64 M	Endocarditis and root abcess	-	Mechanical aortic valve	Surgery Vancomycin Gentamycin Rifampin	Recovered
Liu et al., 2012 [16]	53 M	Endocarditis	-	Bioprosthetic aortic valve	Daptomycin	Recovered

**Table 4:** Orthopedic cases.

Case	Age/Sex	Condition	Underlying Disease	Implanted Device	Definitive Therapy	Outcome
Chan et al., 2012 [17]	45 M	Artificial tissue infection	-	Silicon tendon graft	Surgery Doxycycline	Recovered
Jacquier et al., 2012 [18]	50 F	Artificial discitis	Diabetes Mellitus type 2	Prosthetic L3-L4 disc	Ofloxacin Rifampin	Recovered
Shah et al., 2017 [19]	80 F	Orthopedic implant infection	Chronic adrenal insufficiency	Intermedullary nail	Surgery Daptomycin	Recovered

**Table 5:** Pediatric with implants cases.

Case	Age/Sex	Condition	Underlying Disease	Implanted Device	Definitive Therapy	Outcome
Jourdain et al., 2009 [20]	13 m M	Implant device infection	Hydrocephalus	VPS (ventriculoperitoneal shunt)	Surgery Vancomycin Meropenem Rifampin	Recovered
Schaumburg et al., 2013 [21]	3.9 F	Implant device infection	Ganglioma	VPS	Surgery Cefuroxime Gentamycin	Recovered
Bayraktar et al., 2018 [22]	3 M	Implant device infection	Congenital Adrenal Hyperplasia	VPS	Vancomycin	Deceased

## Data Availability

Not applicable.
